# Effectiveness of circular external fixator in periprosthetic fractures around the knee

**DOI:** 10.1186/s12891-020-03352-9

**Published:** 2020-05-21

**Authors:** Koji Nozaka, Naohisa Miyakoshi, Michio Hongo, Yuji Kasukawa, Hidetomo Saito, Hiroaki Kijima, Hiroyuki Tsuchie, Motoki Mita, Yoichi Shimada

**Affiliations:** Department of Orthopedic Surgery, Akita University Graduate School of Medicine, 1-1-1 Hondo, Akita, 010-8543 Japan

**Keywords:** Circular external fixation, Periprosthetic fractures around the knee, Union rate, Walking level, Closed reduction technique

## Abstract

**Background:**

The incidence of periprosthetic fractures after total joint arthroplasty (TJA) is rising due to an increasing number of TJAs performed annually and the growing elderly population. In many elderly patients with periprosthetic fractures, the bone strength is lowered due to the deterioration of bone quality and a decrease in bone quantity; rigid fixation of the fracture is difficult. It is a challenging operation for orthopedic surgeons. The usefulness of circular external fixation for periprosthetic fractures has been reported in several case studies. The aim of this study was to investigate the rate of union and complications associated with circular external fixation in periprosthetic fractures around the knee.

**Methods:**

We included 19 patients with periprosthetic femur and tibial fractures who underwent osteosynthesis using a circular external fixator and had at least 2 years of follow-up. All patients had comorbidities and high risks associated with anesthesia. Tourniquets were not used in any of the patients. There were no cases in which the skin incision was placed, and the closed reduction technique was used in all cases.

**Results:**

A 100% union rate was achieved with no serious complications. All fractures healed after a mean time of 14.3 ± 5.2 weeks (range, 8–38 weeks). The walking ability was the same level as before the injury in 13 cases.

**Discussion:**

There are many comorbidities associated with periprosthetic fractures in elderly patients. Double-plate or revision surgery were largely invasive and had high risks associated with anesthesia. Circular external fixation is a feasible and effective treatment option because it provides stable fixation, prompt postoperative mobilization, and has no major complications, especially in elderly patients who are treated for periprosthetic fractures.

**Conclusion:**

Circular external fixation is a safe and reliable method for periprosthetic fractures around the knee in elderly patients.

**Level of evidence:**

Level IV, retrospective case series.

## Background

The incidence of periprosthetic fractures after total joint arthroplasty (TJA) is rising due to an increasing number of TJAs performed annually and the growing elderly population. With an increase in the number of elderly patients with osteoporosis, artificial femoral head replacement (FHR) is also emerging as a simple procedure for femoral neck fractures [1, 2]. Furthermore, because total hip arthroplasty (THA) and total knee arthroplasty (TKA) generally result in prolonged use of the artificial joint, the incidence of periprosthetic fracture also increases in elderly patients after TJA and FHR. Periprosthetic fractures often result from minor trauma [1], which may be due to inappropriate placement of the components during surgical procedures (i.e. femoral notch), sex-related differences, osteoporosis, rheumatoid arthritis, neurological diseases, steroid use, or stress shielding [3, 4]. In many elderly patients with periprosthetic fractures, the bone strength is lowered due to the deterioration of bone quality and a decrease in bone quantity; rigid fixation of the fracture is also difficult [5]. The union rate of periprosthetic fracture ranges from 60 to 70%, according to previous literature [6, 7], and it is a challenging operation for orthopedic surgeons [4]. Because there are many elderly patients with periprosthetic fractures, it is important to establish minimally invasive surgical techniques for treatment and promote early rehabilitation. In some cases, conservative treatment is chosen because there are high risks associated with anesthesia or surgery is otherwise difficult to perform. However, in such cases, a long-term non-weight bearing period is necessary for effective healing. Furthermore, the *union* rate of conservative treatment for periprosthetic fractures around the knee is not high [8, 9]. Few patients are capable of restoring their walking ability, even after the long-term non-weight bearing period, which is required for conservative treatment. Circular external fixation is advantageous because it ensures rigid fixation through the insertion of several thin 1.8-mm wires. Furthermore, immediate full weight-bearing after surgery is possible because of rigid fixation.

In addition, by using ligamentotaxis, reduction is possible from the outside of the body, even in large displacement fractures. Osteosynthesis can be completed using a minimally invasive surgical technique and does not require exfoliation or incision of the skin and/or muscle around the fracture site. The usefulness of circular external fixation for periprosthetic fractures has been reported in several case studies [10–13]. In addition, the Ilizarov technique is an effective treatment method for complex limb injuries that is especially applicable in developing or poor countries, and furthermore, in secondary care public hospitals with limited resources [14].

The aim of this study was to investigate the rate of union and complications associated with circular external fixation among elderly patients with periprosthetic fractures.

## Methods

**Fig. 1:**
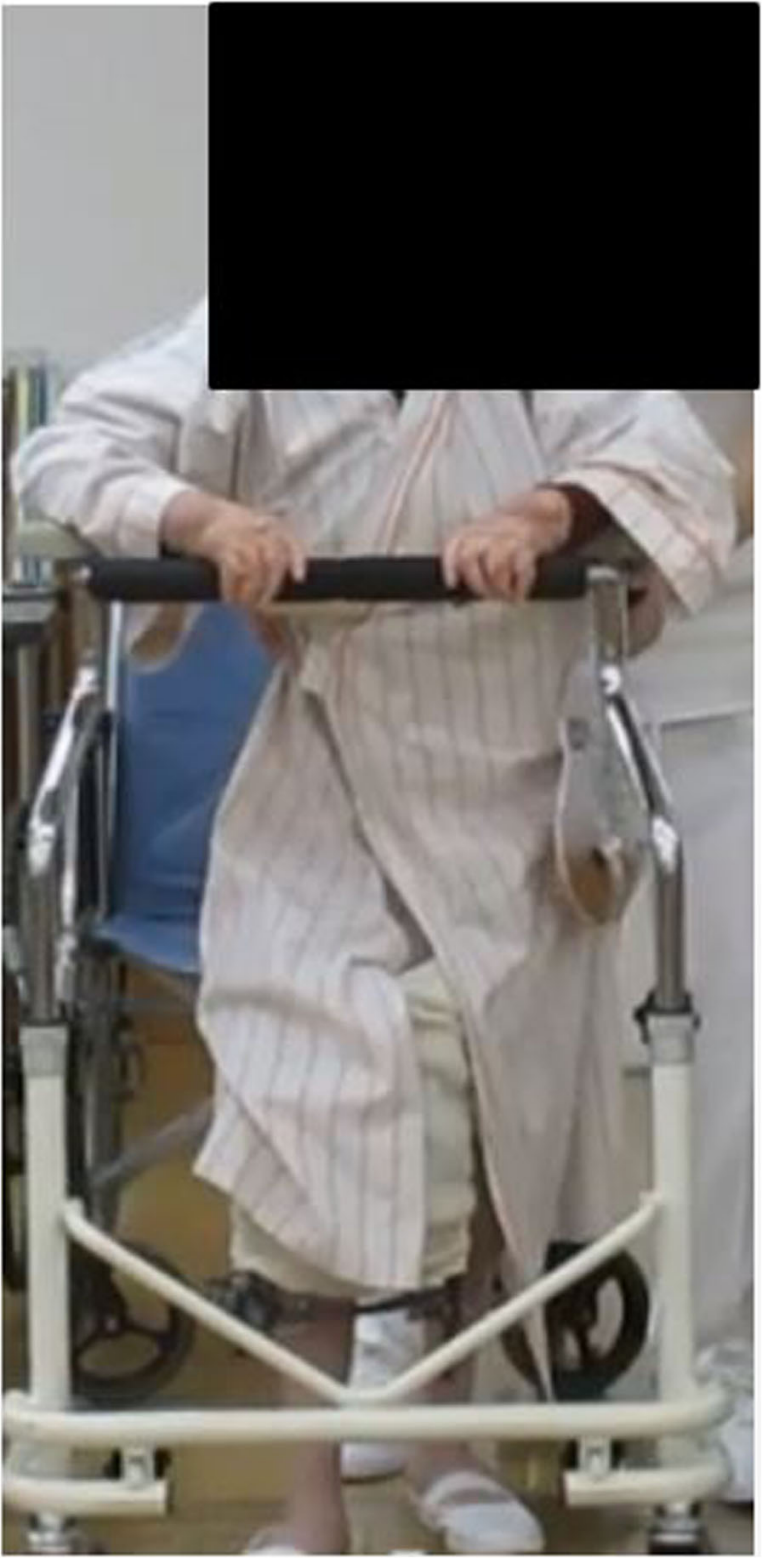
Full weight-bearing walking immediately after surgery

**Fig. 2:**
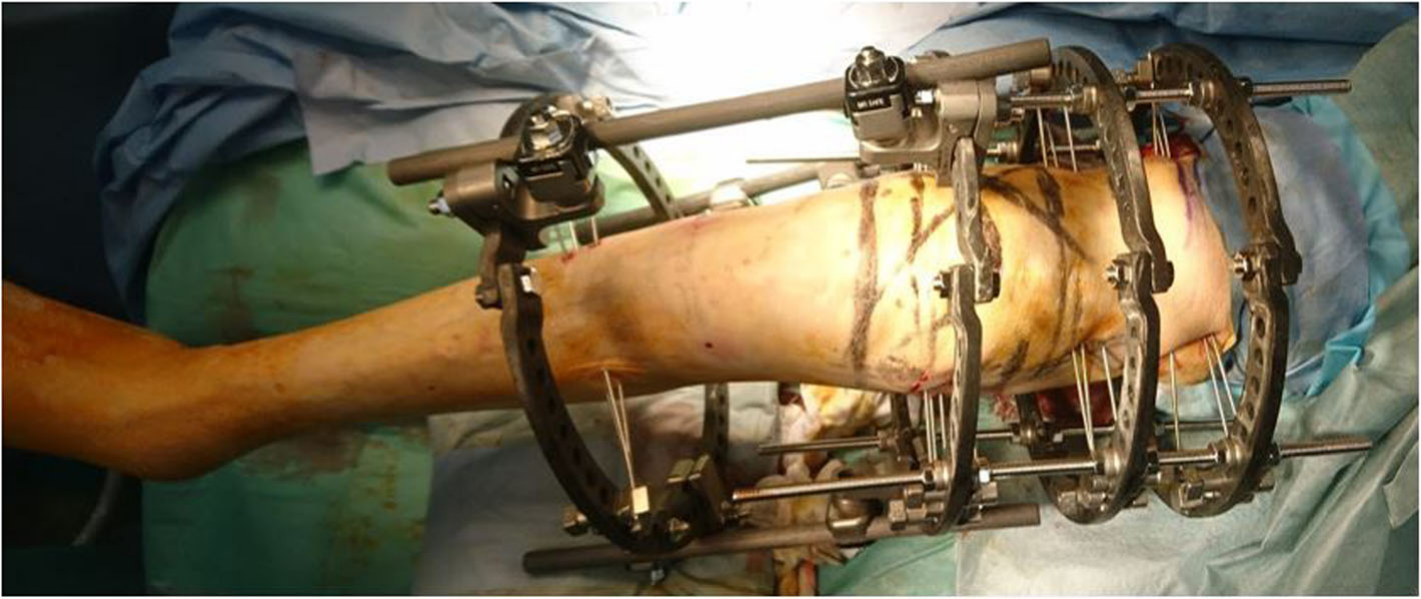
The knee joint-spanning circular external fixator was used, the tibial ring was removed 2 weeks after surgery, and range of motion (ROM) exercises were started

We included 19 patients (4 males and 15 females; mean age: 79.2 years; age range: 60–88 years) with periprosthetic femur and tibial fractures who underwent osteosynthesis using a circular external fixator and had ≥2 years of follow-up. Eleven patients were classified with Rorabeck type II fractures [15], 6 were classified with Vancouver type C fractures, and 1 had a periprosthetic tibial fracture classified according to previously described criteria [16]. One patient experienced periprosthetic fracture between THA and TKA. All periprosthetic fractures were caused by low-energy trauma and the left side was affected in 10 patients. All patients had comorbidities (Table 1). Three patients (15.8%) were receiving treatment for osteoporosis at the time of admission. All patients had low activity levels; for example, four patients walked with rollator, eight patients walked with the assistance of a T-cane, and seven patients were able to walk with no assistance. In all the patients, the anatomical axis of the lower limb was examined before the injury and after surgery. Postoperative bone mineral density (BMD) was measured in 14 patients, and the average BMD of the femur was 0.367 ± 0.028 g/cm^2^. Surgery was requested due to the difficulty associated with inserting an intramedullary nail. Moreover, double-plate or revision surgery were largely invasive and had high risks associated with anesthesia. Therefore, a circular external fixator was used in all patients. Tourniquets were not used in any patients. Skin incisions were not used in any cases, and all cases were reduced using the closed reduction technique. All patients were allowed to walk with full weight-bearing immediately after surgery (Fig. 1). The knee joint-spanning external fixator was used (Fig. 2), the tibial ring (or femur ring; case 19) was removed 2 weeks after surgery, and *range of motion (*ROM) exercises were started. All patients were examined for pin-tract infection by using erythrocyte sedimentation rate (ESR) and C-reactive protein (CRP) levels as common inflammatory markers. ESR and CRP levels were measured every 2 weeks after surgery [17].

**Table 1:** Table 1

Case no.	Sex/age (years)	Follow-up (months)	Comorbidities	Vancouver classification	Lewis–Rorabeck classification	Felix classification type	Time to union (weeks)
1	F/87	54 (died)	Rheumatoid arthritis, Untreated osteoporosis	C			15
2	M/80	72	Chronic heart failure, Chronic renal failure, Untreated osteoporosis	C			11
3	F/69	89	Hypertension, Untreated osteoporosis	C			12
4	M/69	73	Hypertension, Diabetes mellitus	C			20
5	F/81	50	Hypertension, Diabetes mellitus, osteoporosis				10
6	F/87	88	Hypertension, Diabetes mellitus, Untreated osteoporosis		II		12
7	F/83	85	Hypertension, Untreated osteoporosis		II		12
8	F/80	49	Diabetes mellitus, Untreated osteoporosis		II		11
9	F/88	52	Hypertension, Untreated osteoporosis		II		11
10	F/81	69	Hypertension, Diabetes mellitus, Chronic heart failure, Untreated osteoporosis		II		8
11	F/87	51	Hypertension, Diabetes mellitus, Chronic heart failure, Untreated osteoporosis		II		12
12	F/84	16	Hypertension, Chronic heart failure, Untreated osteoporosis		II		11
13	F/68	89	Hypertension, Diabetes mellitus, Untreated osteoporosis		II		15
14	F/79	75	Untreated osteoporosis		II		10
15	M/64	57	Diabetes mellitus		II		27
16	M/70	68	Diabetes mellitus		II		11
17	F/64	24	Rheumatoid arthritis, Hypertension, Osteoporosis		II		12
18	F/84	70	Severe anemia, Severe obesity, Hypertension, Diabetes mellitus, Osteoporosis		II		38
19	F/84	19 (died)	Rheumatoid arthritis, Hypertension, Diabetes mellitus, Untreated osteoporosis			IIA	10
Mean	79.2	62.8					14.1

F, female; M, male

### Surgical technique for periprosthetic femur fracture

First, two straight wires were inserted into the proximal-to-mid femur and attached to the proximal full ring. Then, two straight wires were inserted into the proximal tibia and attached to the ring. The middle 2 full rings were left free near the proximal ring so that they did not interfere when checking the reduction of the fracture area. An assistant held the proximal ring while the surgeon moved the distal tibia ring, which was anchored to the proximal tibia during distraction, flexion, extension, valgus, and varus. This maneuver was gently and carefully repeated over time. By relieving the “jamming” of the fracture area by longitudinal traction with a large force using the tibial ring, almost all dislocations (shortening, rotational, angular, axial) were accurately reduced by closed manipulation. A 2.4-mm Kirschner wire (K-wire) was inserted and fixed to the cross from the inside and outside of the distal fragment. The thin K-wires were inserted in the thick parts of the cortical bone as accurately as possible to prevent cutting of the osteoporotic bone [18]. In some cases, we performed the Kapandji K-wiring technique. We achieved closed reduction in all cases [18]. In addition, the surgeon used the olive wire technique to further reduce the dislocation. The middle 2 rings were anchored using straight wires. Finally, the parts of the rods that protruded distally were cut so that they did not interfere when checking the reduction.

## Results (Table 1, Figs. 3, 4, 5)

A 100% union rate was achieved with no serious complications. All fractures healed after a mean time of 14.3 ± 5.2 weeks (range, 8–38 weeks). One patient had delayed union at 38 weeks. The mean follow-up period was 62.1 ± 12.2 months (range, 13–89 months). Three patients died from heart failure. The mean ROM after follow-up was 110°. Most of the patients felt that their knee flexion was restricted after surgery, compared to before the injury. The walking ability was the same level as before the injury in 13 patients, and 6 patients experienced a lowered walking ability after the surgery. None of the patients in this study showed a change in the anatomical axis of the lower limb after surgery as compared with before the injury. There were fourteen superficial pin-tract infections, which were treated with empirical oral antibiotics and daily pin-tract dressings. Nerve palsy, deep infection, deep venous thrombosis, or pulmonary embolism did not occur in any patients.

**Fig. 3:**
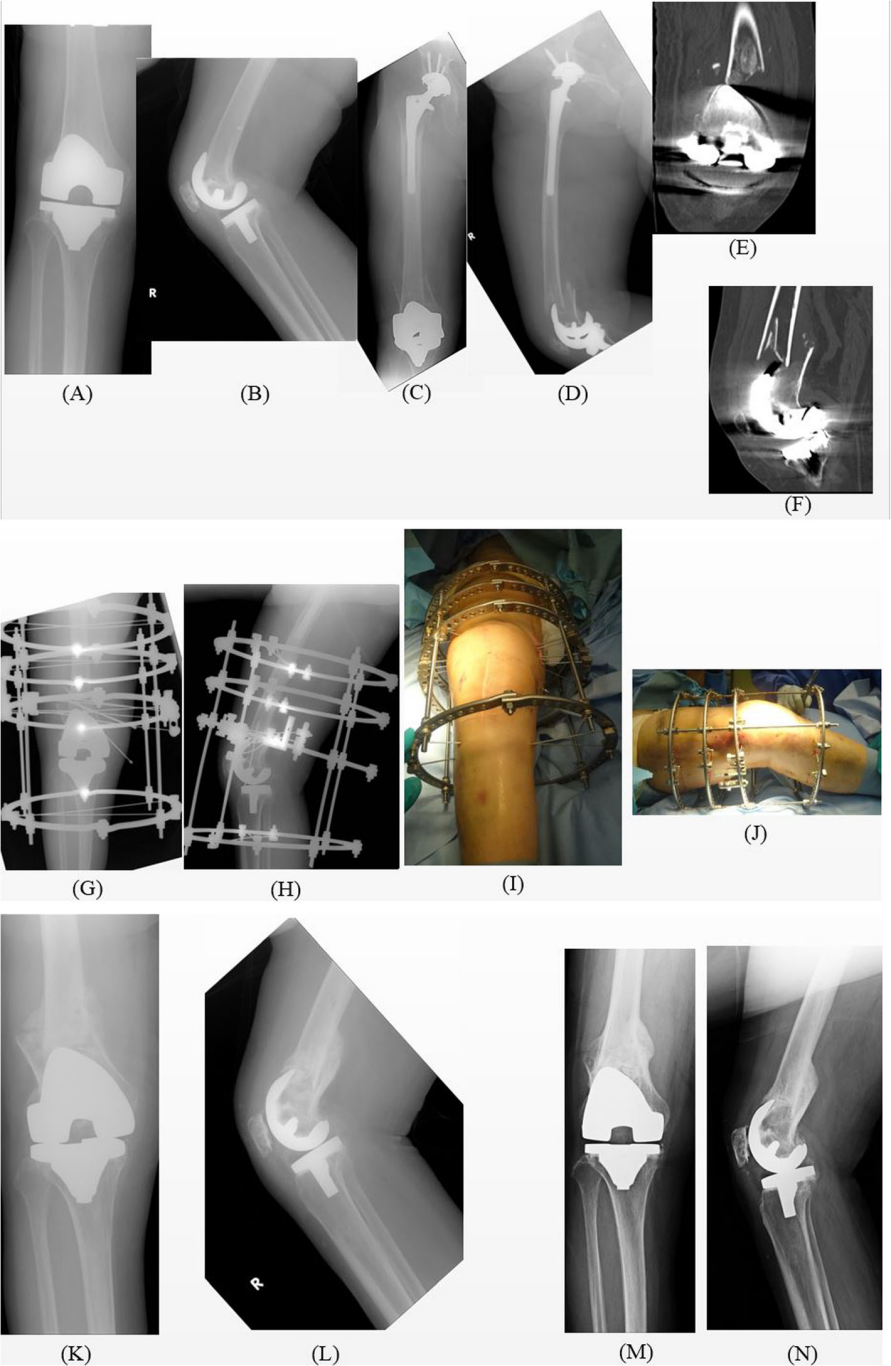
Periprosthetic distal femur fracture in case number 5 treated with a circular external fixator. (A, B) The joint before injury. (C) Preoperative antero-posterior X-ray after the injury. (D) Preoperative lateral angle X-ray. (E) Preoperative coronal CT scan. (F) Preoperative sagittal CT scan. (G, H) Immediate postoperative X-ray. (I, J) Immediate postoperative clinical photograph. (K, L) X-ray after removal of the circular external fixator and weight-bearing (10 weeks after the surgery). (M, N) Three years after the surgery. CT, computed tomography

**Fig. 4:**
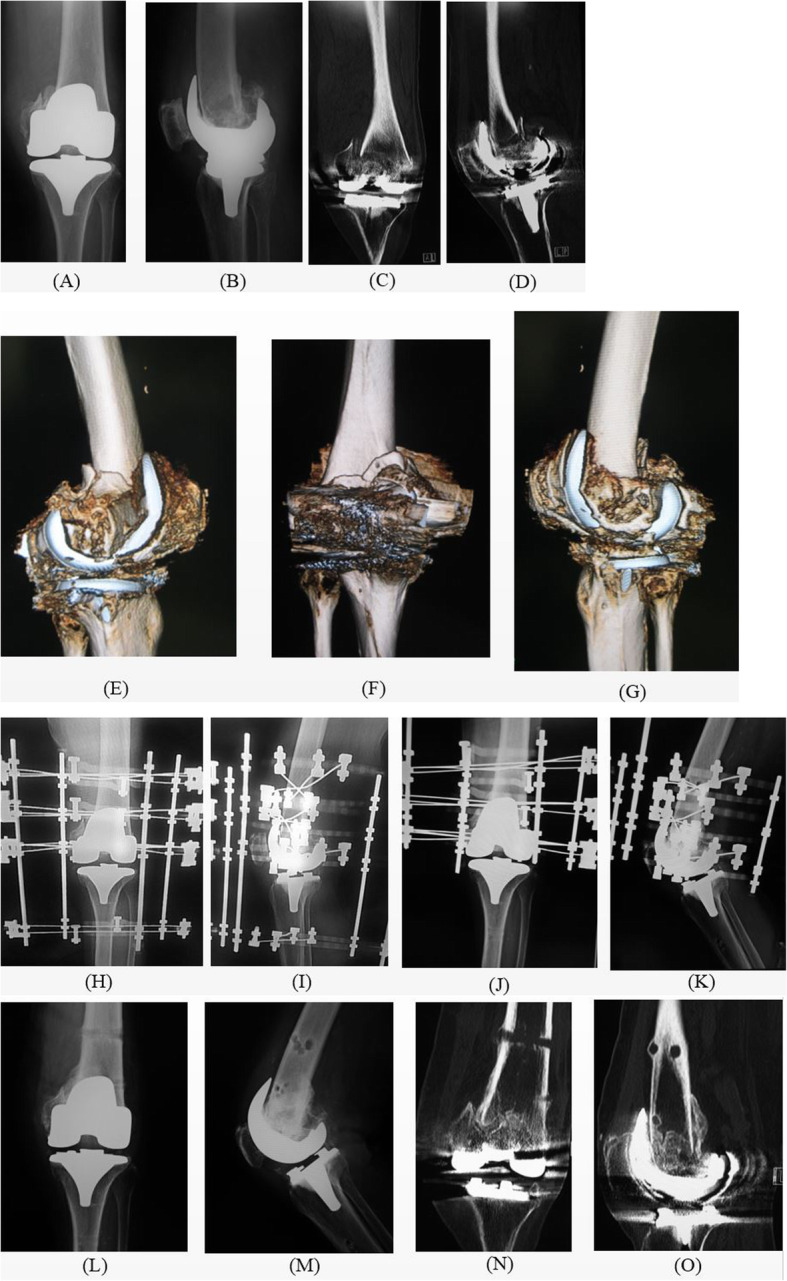
Periprosthetic distal femur fracture in case number 11 treated with a circular external fixator. (A) Preoperative antero-posterior X-ray. (B) Preoperative lateral angle X-ray. (C) Preoperative coronal CT scan. (D) Preoperative sagittal CT scan. (E) Preoperative medial-lateral angle 3D CT. (F) Preoperative posterior-anterior 3D CT. (G) Preoperative lateral-medial angle 3D CT. (H, I) Immediate postoperative X-ray. (J) Antero-posterior X-ray 2 weeks after removal of the tibial ring. (K) Lateral angle X-ray 2 weeks after removal of the tibial ring. (L,M) X-ray after circular external fixator removal and weight-bearing, postoperative 12 weeks. (N,O) CT after circular external fixator removal and weight-bearing, postoperative 12 weeks. CT, computed tomography; 3D, three-dimensional

**Fig. 5:**
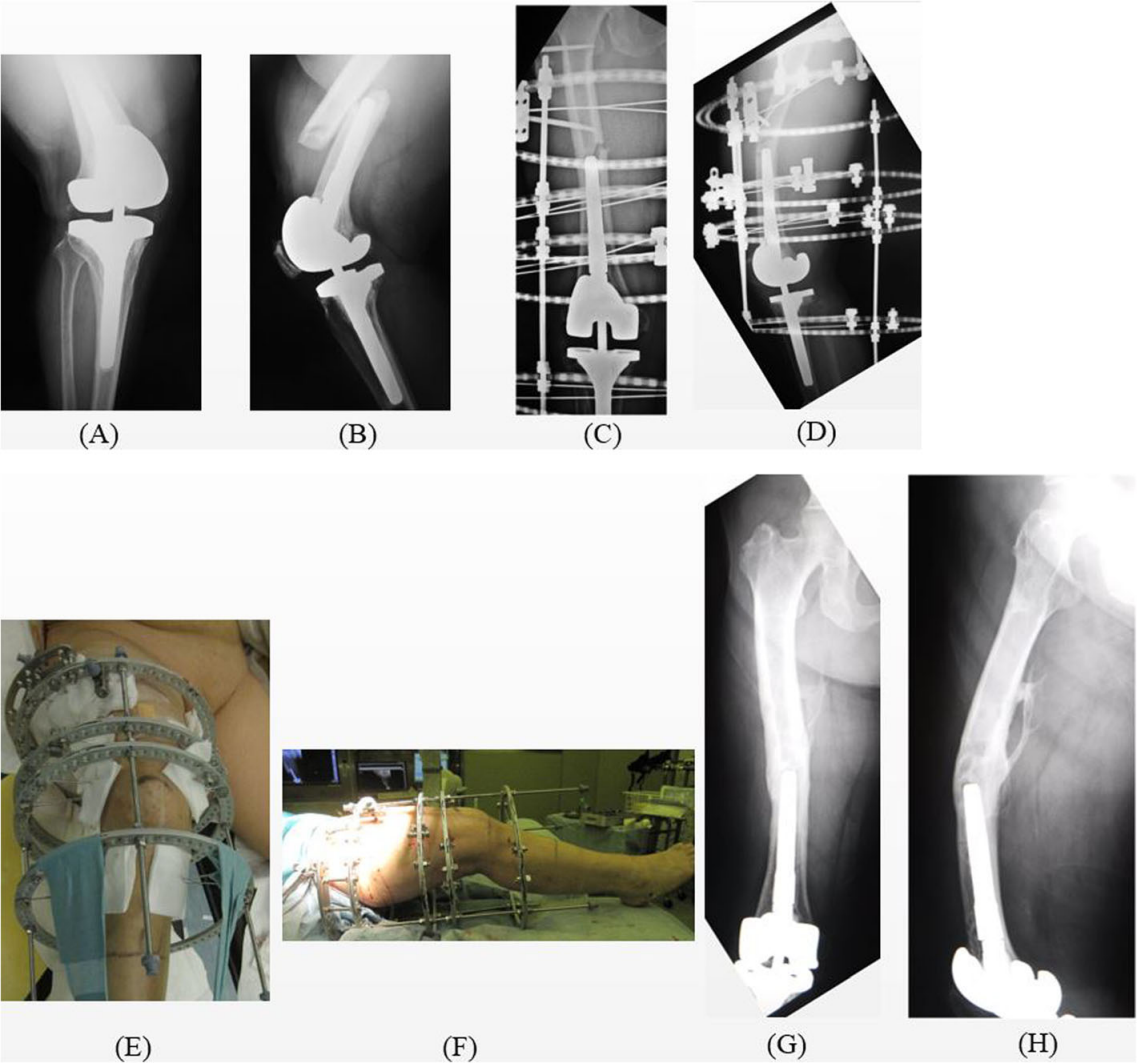
Periprosthetic distal femur fracture in case number 18 treated with a circular external fixator. (A) Preoperative antero-posterior X-ray. (B) Preoperative lateral angle X-ray. (C, D) Immediate postoperative X-ray. (E, F) Immediate postoperative clinical photograph. (G, H) Postoperative 5 years

## Discussion

The incidence of post-TKA supracondylar femoral fracture ranged from 0.3 to 2.5% in patients who had undergone TKA [19], while the incidence of post-THA peri-stem fracture ranged from 1 to 7.8% in patients who had undergone THA [20]. While periprosthetic fracture of the proximal tibia following TKA is rare, it may create a challenging clinical scenario. Although this fracture rarely occurs, periprosthetic fractures around the knee are usually difficult to treat. At present, the most commonly used materials for osteosynthesis are also used for internal fixation of fractures [21]. Surgical treatment is required for periprosthetic fractures with displacement, and a locking plate or retrograde intramedullary nail is often chosen. Using a retrograde intramedullary nail without exfoliation around the fracture site is advantageous for bone union because it allows preservation of the periosteum and peripheral soft tissue. However, the nail cannot be used in patients with severe knee contracture, patients with ipsilateral THA or FHR, or patients without intercondylar space due to TKAs (stem implants or closed box-type femoral components). The distal fragment must be large enough to allow the insertion of many screws so that adequate fixation can be achieved. When a locking plate is chosen for the fragile bone in periprosthetic fractures around the knee, rigid fixation is possible because several screws can be inserted in the distal bone fragment. Rollo et al. reported that plating and bone grafting may ensure better mechanical and biological support for the healing of periprosthetic fracture of the knee than simple plating [22]. In cases of severe osteoporosis and comminuted fracture of the medial metaphysis, a double plate may be preferable. Considering the reduced bone union ability in the elderly, the minimally invasive plate osteosynthesis (MIPO) method, in which the soft tissue is largely exfoliated and the blood flow is not decreased, is expected to promote bone union. However, the MIPO method is limited to cases in which the dislocation of fracture site is very small [1]. It is necessary to adequately expand or reduce the fracture site for cases in which the amount of preoperative fracture dislocation (shortening, rotational, angular, axial) is large. In addition, when the double-plate is used to increase the fixation, the surgery becomes more invasive. Matlovich et al. compared 38 patients who received a locking plate with 19 patients who received an intramedullary nail and reported no significant differences regarding the fusion time or postoperative outcomes [23]. Both retrograde intramedullary nailing and plate fixation require non-weight bearing for approximately 4 to 6 weeks after surgery. Most cases of periprosthetic fracture in elderly patients are associated with poor motor function and difficulty in walking with crutches; therefore, it is difficult to recover the *walking ability* of patients with *disuse syndrome, which is commonly caused by* non-weight bearing for a long time after treatment.

There are many comorbidities associated with periprosthetic fractures in elderly patients; there were only a few cases in this study in which the patient did not have a low activity level or difficulty walking. In patients with *high* preoperative risks, conservative treatment is chosen. It is very difficult for elderly patients to walk again after long-term non-weight bearing. In addition, many patients who undergo internal fixation for periprosthetic fracture can touch their toes and withstand 1/3 full weight-bearing 2–4 weeks postoperatively, and full weight-bearing by 4–8 weeks postoperatively. On the other hand, most patients receiving circular external fixation in this study were able to withstand partial weight-bearing 1 day postoperatively and full weight-bearing 2 weeks postoperatively.

Some reports have indicated that a major advantage of circular external fixation is the ability to achieve rigid fixation for osteoporotic bones, which can be obtained through the insertion of multiple thin, straight wires [10]. Beris et al. reported that circular external fixation is a feasible and effective treatment option because it provides stable fixation, prompt postoperative mobilization, and has no major complications, especially in elderly patients who are treated for periprosthetic fractures [10]. Furthermore, gentle closed reduction and fixation are beneficial for effective bone union, in terms of biological characteristics and vascularization of the fracture area [24]. In addition, none of the patients showed any change in the anatomical axis of the lower limb after surgery in this study. Falzarano et al. reported no significant difference in the anatomical axis of the non-articular tibial fracture between the hybrid external, plate and screw, and intramedullary nailing fixation groups [25].

This approach carries a risk of pin-tract infections. When treating periprosthetic fractures around the knee with a circular external fixator, meticulous pin care and immediate treatment with antibiotics are necessary at any sign of infection [18, 26–32]. Falzarano et al. reported that ESR and CRP levels proved to have greater diagnostic accuracy in predicting late chronic and early postoperative infections in THA. These markers are valuable support for the surgeon in monitoring early postoperative superficial pin-tract infection of the circular external fixator in periprosthetic fractures around the knee. We conducted postoperative evaluation for the prevention of pin-tract infection, using ESR and CRP levels as common inflammatory markers [17].

This study has some limitations that should be addressed. First, patients may find the use of a circular external fixator uncomfortable. However, immediate full weight-bearing after surgery is possible because of rigid fixation. Secondly, most of the patients felt that their knee flexion was more restricted after the surgery than before the injury. Additional studies with a larger number of elderly patients with periprosthetic fractures around the knee are needed to confirm the use of a circular external fixator as a feasible and effective treatment option.

## Conclusions

At the hands of an expert, circular external fixation is a minimally invasive surgical technique that can be performed rapidly without major complications. Our study indicates that circular external fixation is a safe and reliable method for treating periprosthetic fractures around the knee in elderly patients.

## Data Availability

The datasets used and/or analyzed during the current study are available from the corresponding author on reasonable request.

## Abbreviations

TJA: Total joint arthroplasty
FHR: Femoral head replacement
THA: Total hip arthroplasty
TKA: Total knee arthroplasty
BMD: Bone mineral density
ROM: Range of motion
ESR: Erythrocyte sedimentation rate
CRP: C-reactive protein
MIPO: Minimally invasive plate osteosynthesis
